# Supplementary material for: Effects of a Low-Fat Vegan Diet on Gut Microbiota in Overweight Individuals and Relationships with Body Weight, Body Composition, and Insulin Sensitivity. A Randomized Clinical Trial
Source: Nutrients. 2020 Sep 24;12(10):2917. doi: 10.3390/nu12102917 (PMC7598634; doi:10.3390/nu12102917)
Supplement: Supplementary file 1 [file nutrients-12-02917-s001.pdf]

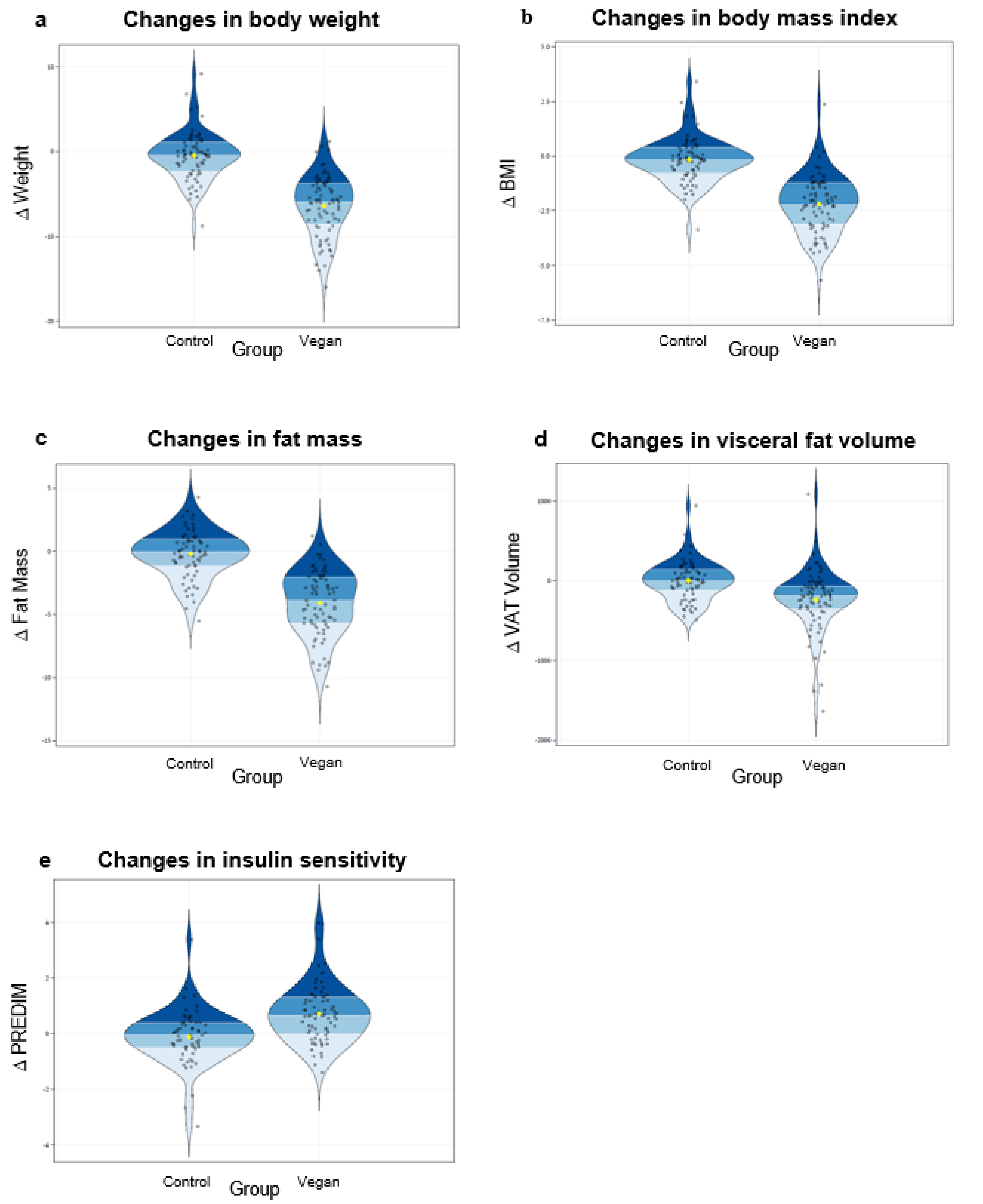

Supplemental Figure 1. Distributions of changes in metabolic outcomes in both groups in the form of violin plots. In these plots, all individual data points are made visible by adding a small random quantity to each. The median of the distribution is shown by the yellow diamond, and the quartiles of the distribution are shown by bands of progressively darker blue shading.

A: Changes in body weight; B: Changes in body mass index; C: Changes in fat mass; D: Changes in visceral fat volume; E: Changes in insulin sensitivity.

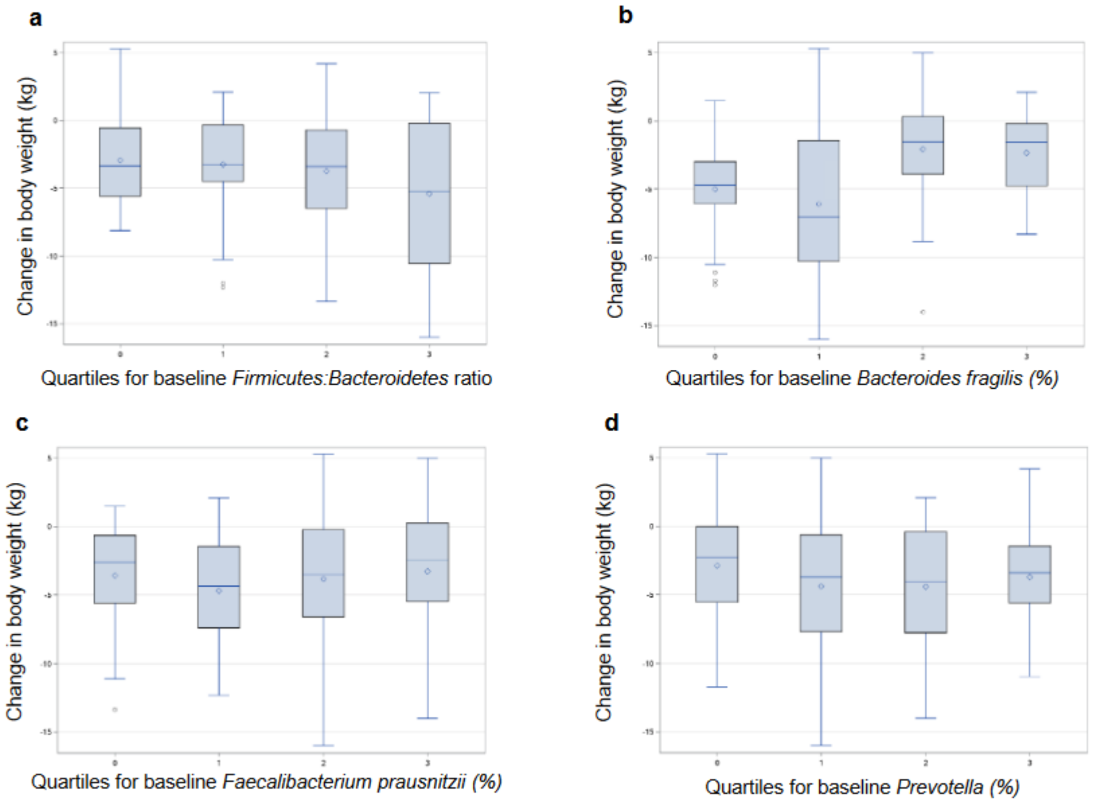

Supplemental Figure 2. Relationship between changes in body weight and quartiles of baseline Firmicutes:Bacteroidetes ratio; F=2.19; p=0.09 (a), relative abundance of *Bacteroides fragilis;* F=8.05; p<0.001 *(b)*, *Faecalibacterium prausnitzii;* F=0.65; p=0.59 (c), and *Prevotella;* F=0.91; p=0.44 (d).
